# Geographic distribution of *Angiostrongylus cantonensis* in wild rats (*Rattus rattus*) and terrestrial snails in Florida, USA

**DOI:** 10.1371/journal.pone.0177910

**Published:** 2017-05-18

**Authors:** Heather D. Stockdale Walden, John D. Slapcinsky, Shannon Roff, Jorge Mendieta Calle, Zakia Diaz Goodwin, Jere Stern, Rachel Corlett, Julia Conway, Antoinette McIntosh

**Affiliations:** 1 Department of Infectious Disease and Pathology, University of Florida College of Veterinary Medicine, Gainesville, Florida, United States of America; 2 Florida Museum of Natural History, University of Florida, Gainesville, Florida, United States of America; University of Pretoria, SOUTH AFRICA

## Abstract

The parasitic nematode *Angiostrongylus cantonensis* is a major cause of eosinophilic meningitis in humans, and has been documented in other incidental hosts such as birds, horses, dogs and non-human primates. It is endemic in Hawaii, and there have been sporadic reports in the southern continental United States. This parasite uses rats as definitive hosts and snails as intermediate hosts. In this study, we collected potential definitive and intermediate hosts throughout Florida to ascertain the geographic distribution in the state: Rats, environmental rat fecal samples, and snails were collected from 18 counties throughout the state. Classical diagnostics and morphological identification, along with molecular techniques were used to identify nematode species and confirm the presence of *A*. *cantonensis*.

Of the 171 *Rattus rattus* collected, 39 (22.8%) were positive for *A*. *cantonensis*, and 6 of the 37 (16.2%) environmental rat fecal samples collected in three of the surveyed counties were also positive for this parasite by real time PCR. We examined 1,437 gastropods, which represented 32 species; 27 (1.9%) were positive for *A*. *cantonensis* from multiple sites across Florida. Three non-native gastropod species, *Bradybaena similaris*, *Zachrysia provisoria*, and *Paropeas achatinaceum*, and three native gastropod species, *Succinea floridana*, *Ventridens demissus*, and *Zonitoides arboreus*, which are newly recorded intermediate hosts for the parasite, were positive for *A*. *cantonensis*. This study indicates that *A*. *cantonensis* is established in Florida through the finding of adult and larval stages in definitive and intermediate hosts, respectively, throughout the state. The ability for this historically subtropical nematode to thrive in a more temperate climate is alarming, however as the climate changes and average temperatures rise, gastropod distributions will probably expand, leading to the spread of this parasite in more temperate areas. Through greater awareness of host species and prevalence of *A*. *cantonensis* in the United States, potential accidental infections may be avoided.

## Introduction

*Angiostrongylus cantonensis*, the rat lungworm [1], is a parasitic metastrongyloid nematode that is a major cause of eosinophilic meningitis in humans [2–5], and has been described in over 2800 documented cases in more than 30 countries worldwide [4, 6]. The lifecycle of this parasite has been well described [5, 7–8], and is indirect, requiring multiple hosts for completion. The definitive hosts include several species of rodents, commonly *Rattus* spp., which ingest the infective third stage larvae (L3) of this parasite found in gastropod intermediate hosts. In some instances, paratenic hosts, such as frogs, crustaceans or fish harbor the infective L3s, and serve as a source of infection, as well [9–13]. Once the L3s are ingested by the rat, they travel passively in the bloodstream before entering the central nervous system and develop to an immature adult. They then re-enter the circulatory system and travel to the pulmonary artery where they become sexually mature. Females lay eggs, which larvate and hatch in the lung tissue, and first stage larvae (L1) are coughed up, swallowed, and shed in feces. Gastropods then acquire the L1 that in turn develop to the L3 and continue the cycle. Infections in humans, non-human primates and other animals such as dogs, birds, horses and opossums have been reported [6, 14–21]. These hosts are incidental hosts in which the infective L3s arrest development in the central nervous system. The arrested L3s are unable to develop a patent infection, and subsequent eosinophilic meningitis develops. Both paratenic and incidental hosts become infected through ingesting the infective L3 within the gastropod intermediate host. In humans, adults and children present differently when infected with *A*. *cantonensis*. In adults, clinical signs include headache, stiff neck, fever, vomiting and nausea. Paralysis of the face and limbs is also common. In children, nausea, vomiting and fever are more common than headache or stiff neck [4, 6, 22]. In some instances, ocular angiostrongyliasis develops and surgical removal of worms in the eye is necessary [23]. In severe infections, coma or death may occur [24].

*Angiostrongylus cantonensis* is relatively new to the continental United States, and is presumed to have been introduced in the 1980s from rats arriving on ships into New Orleans, Louisiana [11]. Since its introduction, reported cases of angiostrongyliasis in humans include a young boy who ingested a snail in Louisiana [25] and two young children infected in Texas [26]. However, in Hawaii, where the parasite has been endemic for over 50 years, there have been numerous documented human infections often attributed to intentional or accidental ingestion of snails or slugs [27–29]. In Hawaii, there are several gastropod species reported as potential intermediate hosts of *A*. *cantonensis*, including species both native and non-native to the islands [12]. In this recent study [12], over 1200 specimens were examined from collections made throughout the islands, and 6% of non-native gastropods sampled were positive for *A*. *cantonensis* larvae. In the continental United States, studies have found snails infected with *A*. *cantonensis* in Louisiana and southern Florida [20, 30–32] and infected rats also reported in Louisiana [11, 16]. In Florida, infection with *A*. *cantonensis* can be attributed to a non-human primate death in a zoological park and in a privately owned animal, both instances occurring in southern Florida [17, 20]. Other than a few small reports with limited snail species, the extent of the geographic range of *A*. *cantonensis* in Florida was unknown. The goal of this study was to survey multiple counties throughout Florida, sampling both definitive and intermediate hosts, to obtain a better idea of the geographic range for this zoonotic nematode, and determine whether the gastropod hosts were native or non-native to Florida and the United States.

## Methods

### Ethics statement

The animal protocol and sample collection used in this study was approved and performed under the guidelines set forth by the Institutional Animal Care and Use Committee at the University of Florida (Protocol #201608110). We obtained permission from property owners before any sample collection.

### Rat collection and necropsy

During 2013–2015, 171 rats (*Rattus rattus*) were collected from 45 different sites across 6 counties in Florida. In each county, rats were collected from multiple sites. Additionally, 37 environmental rat fecal samples were collected from 24 different sites across 4 counties in Florida. We obtained rats from private facilities through pest control operations that were currently in place, which included bait trapping. Dead rats were collected and stored at -20°C until they could be transported to the parasitology laboratory at the University of Florida, and necropsied. The heart, lungs, brain and colon were removed. The left lung lobes and brain were stored in 10% formalin and prepared for histological sectioning. The heart and right lung lobe were stored in 70% ethanol and examined for the presence of adult *Angiostrongylus cantonensis*. Any adult nematodes were identified based on morphological characteristics and transferred to a glass specimen jar with 70% ethanol. A section of pulmonary artery was saved at -20°C for molecular analysis. The colon was stored in a sealed container at -20°C. Fecal contents were removed from the colon and a fecal sedimentation was performed to detect L1 larvae of *A*. *cantonensis* [33]. Positive rats were identified by the presence of adult nematodes in the pulmonary artery, L1s in the fecal sediment and/or real time PCR positive results from fecal sediment or pulmonary artery tissue. Environmental fecal samples were also evaluated by fecal sedimentation. Fecal sediment from rats and environmental samples was saved in a 2mL microcentrifuge tube at -20°C for molecular analysis.

### Gastropod collection and digestion

A total of 1,437 gastropods were collected from 71 different sites across 18 counties in Florida. Gastropods were identified based on morphological characteristics [34–37] and were represented by 32 species (21 native and 11 non-native). Live gastropods were examined following published digestion protocols [12, 38] with the following modifications: The body was removed from the shell, if present, mantle and foot removed, minced and transferred to 10-50mL of digestion solution, depending on the amount of material. The digestion solution contained 3% pepsin and 0.7% hydrochloric acid and was constantly agitated at room temperature 3 hours to overnight until the majority of tissue dissolved. The digested liquid solution was then placed in a Baermann apparatus lined with a wire mesh with 2 layers of gauze and allowed to stand for 24 hours to allow for nematode larval migration to the neck of the Baermann funnel. The filtered liquid containing any larvae was transferred to a 50mL conical tube and gently centrifuged for 5 minutes to create a soft pellet. Approximately 75% of the supernatant was removed and the remaining liquid and pellet was transferred to a Petri dish and examined under a stereomicroscope for the presence of larvae. After examination, the material was stored in a 2mL microcentrifuge tube at -20°C for molecular analysis. Dead gastropods were removed from the shell, if present, mantle and foot removed, minced and examined under a stereomicroscope for the presence of larvae. This material was also stored in a 2mL microcentrifuge tube at -20°C for molecular analysis.

### Molecular analysis

All samples were processed for real time PCR and verification of the presence of *A*. *cantonensis*. Approximately 25mg of tissue from nematodes collected in the pulmonary artery of infected rats and tissue from the pulmonary artery of all rats was further macerated and lysed for DNA extraction. Additionally, DNA was extracted from digested and macerated material, and any larvae, collected from snail specimens. The DNA extractions were performed using Qiagen DNeasy^®^ Blood and Tissue Kit (Qiagen) according to manufacturer protocol, with the following modifications: Tissue was transferred to 180μL of tissue lysis (ATL) buffer (Qiagen) in a 2mL microcentrifuge tube and incubated at -80°C for 1 h. Samples were then incubated at 56°C for 5–10 min and 20μL of proteinase K (Qiagen) was added and samples were retained at 56°C until the tissue was completely lysed, usually 1–3 h, vortexing every 30 min. The sample was then processed according to manufacturer protocol to completion. Fecal sediments from necropsied rats and environmental fecal samples were also evaluated. The DNA extractions for these samples were performed using ZR Fecal DNA Miniprep™ (Zymo Research, Irvine, CA) according to manufacturer protocol.

A real time PCR assay was performed using a previously published, species specific primer set amplifying the internal transcribed spacer one (ITS1) region of the 18S rDNA [39] AcanITS1F1 (5’-TTCATGGATGGCGAACTGATAG-3’) and AcanITS1R1 (5’-GCGCCCATTGAAACATTATACTT-3’) each at 0.2μM, and probe AcanITS1P1 (5’FAM-ATCGCATATCTACTATACGCATGTGACACCTG-BHQ-1-3’) at 0.05μM in 20μL total volume containing 1X final concentration TaqMan^®^Fast Advanced Master Mix (Applied Biosystems™ ThermoFisher Scientific, Austin, TX). Standard cycling conditions following TaqMan^®^ assay and Applied Biosystems™ 7500 Fast system protocols were 50°C for 2 min, one cycle of polymerase activation at 95°C for 20 s, followed by 40 cycles denaturing at 95°C for 3 s and annealing and extension at 60°C for 30 s. All samples, along with positive and negative controls, were run in duplicate, and the assay was repeated in duplicate for positive or suspect samples.

To verify positives from the real time PCR assay were truly *A*. *cantonensis* adults and/or larvae, each positive sample was retested using the above mentioned species-specific PCR primers, AcanITS1F1 and AcanITS1R1 and standard PCR protocols in a 20μL total volume containing 5X Phire Reaction Buffer (ThermoScientific™ Phire™ Hot Start II DNA Polymerase, ThermoScientific™) at a 1X final concentration, 200μM each 10mM deoxyribose nucleoside triphosphates (dNTPs), and 0.5μM each primer. Standard cycling conditions following Phire™ Polymerase protocols were one cycle of initial denaturation at 98°C for 30 s, 35 cycles of denaturation at 98°C for 5 s, annealing at 60°C for 5 s and extension at 72°C for 10 s, followed by a final extension at 72°C for 1 min. All products producing visualized bands on agarose gel were sequenced and results verified as *A*. *cantonensis*.

## Results

**Fig 1:**
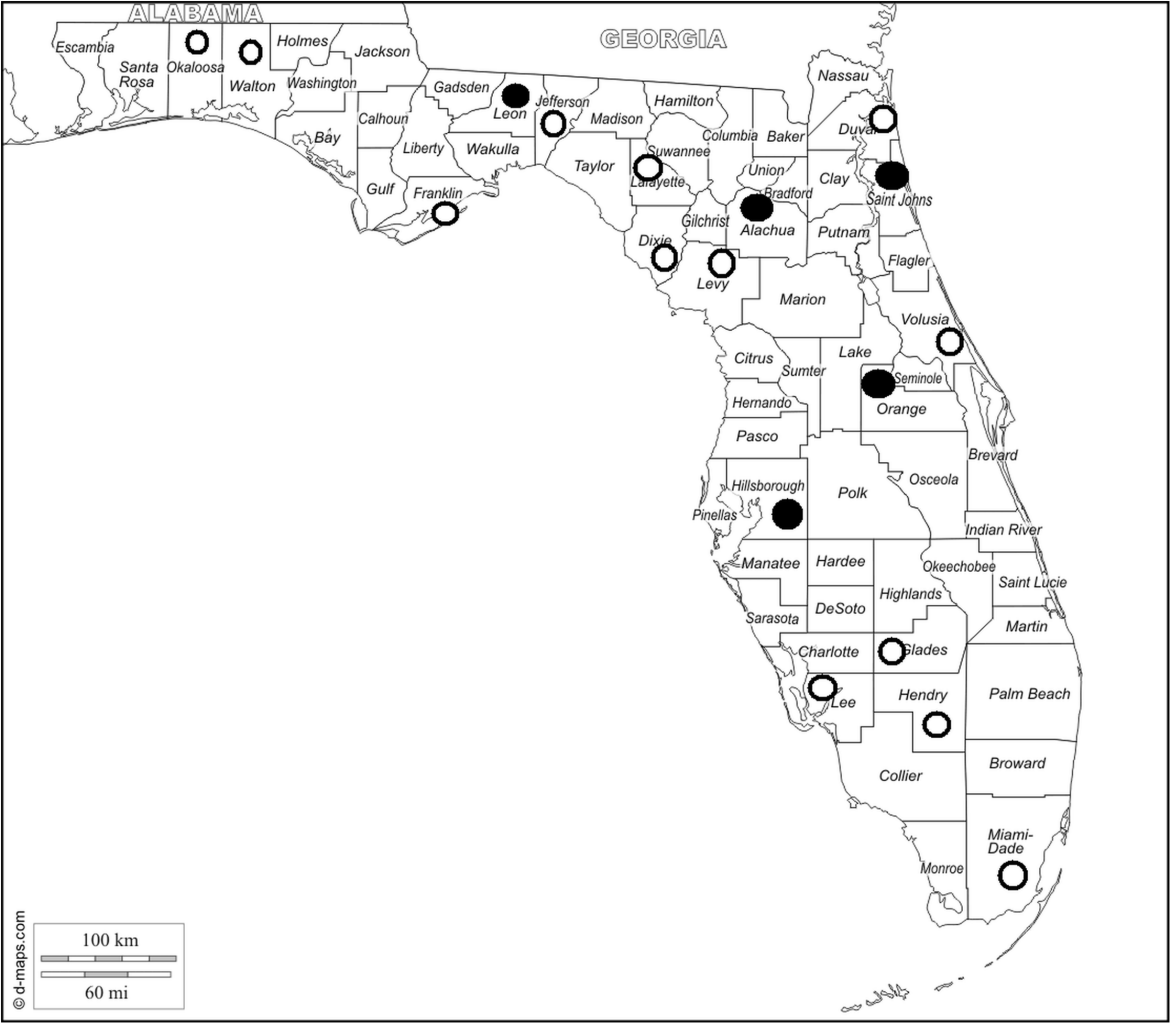
Surveyed counties in Florida, USA, indicating positive and negative areas for *Angiostrongylus cantonensis*. Counties that were positive for *Angiostrongylus cantonensis* from rat, environmental rat feces and/or gastropods samples are marked with closed circles. Counties negative for *A*. *cantonensis* are marked with open circles. The map can be found at http://www.d-maps.com/carte.php?num_car=6867&lang=en.

*Angiostrongylus cantonensis* was found in 5 of the 18 counties sampled in Florida (Fig 1). Of the 171 *Rattus rattus* necropsied, 39 (22.8%) were positive for *A*. *cantonensis* in 3 of 6 counties surveyed and included Alachua, Hillsborough and Orange (Table 1). In 4 counties, environmental rat fecal samples (n = 37) were collected and examined for the presence of L1s. Of those samples, 6 (16.2%) were positive for *A*. *cantonensis* by real time PCR from 3 counties surveyed and included Alachua, Orange and St. Johns (Table 2).

**Table 1:** Rats (*Rattus rattus*) collected and necropsied from multiple sites in 6 counties in Florida.

County	No. rats necropsied	No. rats positive for *A*. *cantonensis* *	No. sites positive per county (total)	No. rats with adult *A*. *cantonensis* nematodes recovered (PCR positive)	Fecal sediments with visualized nematode larvae (PCR positive)	Pulmonary artery tissues PCR positive
Alachua	22	2	1 (6)	1 (1)	1 (0)	2
Duval	7	0	0 (4)	0	0	0
Hillsborough	87	30	18 (25)	24 (24)	11 (19)	23
Okaloosa	7	0	0 (1)	0	0	0
Orange	43	7	2 (5)	3 (3)	6 (3)	7
St. Johns	5	0	0 (4)	0	0	0
Total	171	39	21 (45)	28 (28)	18 (22)	32

* Positive results include rats with adult nematodes recovered, positive PCR results for fecal sediment and/or pulmonary artery tissue.

**Table 2:** Rat environmental fecal samples collected from multiple sites in 4 counties in Florida.

County	No. environmental fecal samples	Fecal sediments with visualized nematode larvae	No. positive for *A*. *cantonensis* (verified by real time PCR)	No. sites positive (total)
Alachua	17	1	3	1 (12)
Duval	11	0	0	0 (3)
Orange	6	4	1	1 (6)
St. Johns	3	0	2	2 (3)
Total	37	5	6	4 (24)

Of the 1,437 gastropods examined, 27 (1.9%) were positive for *A*. *cantonensis* from multiple sites across 4 counties surveyed and included Alachua, Hillsborough, Leon, and Orange (Table 3). Gastropods were represented by 32 species. Of the 21 native species, 3 species were positive for *A*. *cantonensis* and included *Succinea floridana* (3.7% of 27 individuals), *Ventridens demissus* (4.3% of 23 individuals) and *Zonitoides arboreus* (1 of 1 individual). Of the 11 non-native species collected, 3 were positive for *A*. *cantonensis*, *Bradybaena similaris* (2.3% of 939 individuals), *Zachrysia provisoria* (1.2% of 83 individuals) and *Paropeas achatinaceum* (1 of 2 individuals). *Bradybaena similaris* had the highest number of *A*. *cantonensis* infected specimens; however, they were also the most numerous in the survey. *Angiostrongylus cantonensis* was verified in all positive samples and sequences generated from the standard PCR results arranged in a FASTA file (S1 File).

**Table 3:** Gastropod species collected from multiple sites in 18 counties throughout Florida.

County	Gastropod species	No. collected	No. positive for *Angiostrongylus cantonensis*	No. sites positive (total sites)
Alachua	*Allopeas clavulinum*	1	0	0 (1)
	*Bradybaena similaris*	521	12	3 (6)
	*Deroceras leave*	4	0	0 (2)
	*Drymaes dormani*	6	0	0 (1)
	*Gastrocopta* sp.	1	0	0 (1)
	*Mesodon thyroidus*	5	0	0 (1)
	*Ovachlamys fulgens*	1	0	0 (1)
	*Succinea floridana*	21	0	0 (1)
	*Ventridens demissus*	3	0	0 (1)
Dixie	*Daedalochila hausmani*	19	0	0 (1)
	*Euglandina rosea*	2	0	0 (1)
	*Polygyra septemvolva*	8	0	0 (1)
Duval	*Bradybaena similaris*	244	0	0 (13)
Franklin	*Daedalochila subclausa*	4	0	0 (2)
Glades	*Praticolella griseola*	5	0	0 (1)
Hendry	*Praticolella griseola*	6	0	0 (1)
Hillsborough	*Bradybaena similaris*	146	10	2 (3)
	*Ovachlamys fulgens*	3	0	0 (1)
	*Paropeas achatinaceum*	2	1	1 (1)
	*Succinea floridana*	27	1	1 (1)
	*Succinea tenella*	18	0	0 (1)
	*Zachrysia provisoria*	1	0	0 (1)
	*Zonitoides arboreus*	1	1	1 (1)
Jefferson	*Daedalochila hausmani*	2	0	0 (1)
Lafayette	*Daedalochila* sp.	2	0	0 (1)
Lee	*Pomacea maculata*	7	0	0 (1)
Leon	*Ventridens demissus*	20	1	1 (1)
Levy	*Daedalochila auriculata*	2	0	0 (1)
	*Polygyra cereolus*	13	0	0 (1)
	*Polygyra septemvolva*	24	0	0 (1)
	*Melampus bidentatus*	20	0	0 (1)
Miami-Dade	*Bulimulus guadalupensis*	67	0	0 (1)
	*Euglandina rosea*	3	0	0 (1)
	*Subulina octona*	11	0	0 (1)
	*Zachrysia provisoria*	56	0	0 (1)
Okaloosa	*Triodopsis hopetonensis*	18	0	0 (1)
Orange	*Bradybaena similaris*	18	0	0 (3)
	*Lehmannia valentiana*	8	0	0 (1)
	*Melanoides tuberculata*	1	0	0 (1)
	*Planorbella duryi*	2	0	0 (1)
	*Planorbella trivolvis*	1	0	0 (1)
	*Zachrysia provisoria*	26	1	1 (3)
St. Johns	*Bradybaena similaris*	10	0	0 (1)
Volusia	*Daedalochila uvulifera*	2	0	0 (1)
Walton	*Polygyra septemvolva*	72	0	0 (1)
	*Succinea unicolor*	3	0	0 (1)
Total		1437	27	10 (71)*

*Some counties had multiple sites of collection. Gastropod species may have been collected from the same site or multiple sites in the county, indicated by total sites in the final column.

## Discussion

Through this study, we can estimate the geographic range of *Angiostrongylus cantonensis* in Florida, with nematodes recovered from either rats, environmental rat fecal samples and/or gastropods throughout the state. The reality is that this zoonotic nematode is probably more widespread than indicated here, and the extent of intermediate hosts much larger, since many species had few individuals represented in this survey. Gastropods infected with *A*. *cantonensis* included 3 native species and 3 non-native species. The 3 native species that were positive for *A*. *cantonensis*, *Succinea floridana*, *Ventridens demissus* and *Zonitoides arboreus*, are newly recorded intermediate hosts for the parasite and are widely distributed species that are common in anthropogenic habitats. Two of the native species that were positive for *A*. *cantonensis* have ranges that extend well beyond Florida: *V*. *demissus* is widely distributed in the southeastern US and *Z*. *arboreus*, occurs commonly throughout temperate North America [40]. Native species evaluated in this project, which have been shown to serve as intermediate hosts, but were not positive for *A*. *cantonensis* in this study, include *Euglandina rosea*, *Planorbella duryi*, *Mesodon thyroidus* and *Deroceras laeve* [12]. Non-native gastropod species negative for *A*. *cantonensis* in this study but documented as intermediate hosts in other studies were *Lehmannia valentiana*, *Melanoides tuberculata*, *Pomacea maculata*, *Ovachlamys fulgens*, and *Subulina octona* [12]. The non-native gastropods that were positive in this study, *Bradybaena similaris*, *Zachrysia provisoria* and *Paropeas achatinaceum*, have been documented previously as intermediate hosts, and *Z*. *provisoria* specifically from Miami-Dade county in Florida [12, 20, 32, 38]. *Zachrysia provisoria*, introduced to Florida over a century ago, and considered a major pest, was put on a list of quarantine importance in the United States [41]. This voracious feeder, known for damaging ornamental plants [42–43], was recovered as far north as Orange County in this study. *Bradybaena similaris*, collected in multiple counties across Florida, and *P*. *achatinaceum*, from Hillsborough County, are also major, invasive pest species, and found in many areas of the world [44].

Rats of multiple species are known definitive hosts of *A*. *cantonensis* [7–8, 11, 13, 45], however in this study only *Rattus rattus* were collected and examined. Previous studies in Florida [32] have also found environmental rat fecal samples positive for *A*. *cantonensis* in south Florida, specifically Miami (Miami-Dade County). We identified *A*. *cantonensis* in 22.8% of rats tested in Florida, and a recent study in Jamaica, immediately following an outbreak of angiostrongyliasis in humans, found similar prevalence with 32% of all rats sampled infected with *A*. *cantonensis* [46].

Rats are ubiquitous; however, as the climate changes and average temperatures rise, the geographic distributions of gastropod hosts, specifically non-native species, will no doubt expand and lead to the spread of *A*. *cantonensis* into areas with a historically more temperate climate. A model developed by Lv el al. [47] supports this idea and suggests the predicted expansion of *Pomacea canaliculata*, an invasive fresh water snail and important intermediate host of *A*. *cantonensis*, in an increasingly warmer climate will drive the expansion of the endemic area of *A*. *cantonensis* in China. However, a different model suggests that as temperatures increase over time, the range of *A*. *cantonensis* may increase but the variabilities in climate, including humidity and precipitation, may counteract the transmission due to the lifecycle requirements of the parasite [48]. In the United States, transportation of non-native gastropod hosts via plant nurseries has been documented [49–50], and while not every gastropod species transported across state lines, and possibly into a different climate, will survive, many do. Several of the non-native gastropods listed in these studies are reported intermediate hosts for *A*. *cantonensis*, including *B*. *similaris*, *Achatina fulica*, *E*. *rosea and L*. *valentiana*. The movements of these potential hosts from Florida, a state with a large horticultural industry where we now know many sites of *A*. *cantonensis* infection occur, to other states via transportation of plants is a reality that needs attention.

The established area of *A*. *cantonensis* includes the entire state of Florida, and its geographic range is increasing into other states in the southeastern US, through definitive and intermediate hosts. This parasite has been documented in Louisiana in rats and snails since the 1980s [11, 14–16, 30], implicated in the death of a non-human primate in Alabama [51], reported cause of an avian death in California [21], and *A*. *cantonensis* DNA was found in a cotton rat in Oklahoma [52]. Interactions between gastropods and humans and other incidental hosts are increasing. In the continental US, two children presented to a Houston, Texas hospital with fever of unknown origin, later discovered it was caused by *A*. *cantonensis* infection [26]. The children had no known history of travel to endemic areas, no history of gastropod ingestion; however, one child often chewed on lettuce leaves. The authors proposed that recent flooding in the Gulf Coast and Houston area might have increased snail activity and contributed to the infections. In Jamaica, since 2000, there have been 23 reported human cases and food contaminated with snails implicated in over half of these cases [53–54] as none of those infected reportedly ate raw snails. Of those 23 people infected, two infections were fatal and four suffered subsequent neurological damage. Even though there have been no reported human cases of angiostrongyliasis in Florida, since *A*. *cantonensis* is throughout the state, physicians may be misdiagnosing infections due to low numbers of infectious larvae causing minimal disease and the ability to detect worms in infected patients is difficult [4].

Human infections with *A*. *cantonensis* can happen a variety of ways. First, there is knowingly consuming raw or under-cooked snails and slugs that contain the infectious larvae. This is a common source of infection in China and Southeast Asia [55]. Human infections can also occur through accidental ingestion of the infected mollusks. This happens primarily from contaminated vegetables not properly washed, cooked or from the vegetable juice [55]. It has been suggested that mucus secreted from the snail containing infectious L3s [56] and contaminated water [55, 45] may also be potential sources of infection. In the angiostrongyliasis outbreak in Jamaica, it was suggested that cross contamination of produce with infectious larvae could have occurred as street vendors used a single bucket of water to rinse vegetables. These larvae could have come from mucus or feces of snails, or left dead snails [53]. One study demonstrated that infective larvae from *A*. *cantonensis* can contaminate water (i.e. terrestrial snails fall into water and drown) and found that water treated with chlorine or iodine had no effect on the larvae [57]. Thorough washing of produce will effectively remove snails and slugs if done on an individual leaf basis, and that washing with solutions such as bleach, vinegar and salt were just as effective as water [58]. This allows potential accidental infections through inadvertently ingesting small, unnoticed snails, thought to be the most common route of infection in Hawai’i [55]. In addition to infected intermediate hosts, paratenic hosts play an important role in the transmission of *A*. *cantonensis*, with many species recognized throughout the world [55]. Predatory flatworms, specifically *Platydemus manokwari*, ingest infected snails and slugs and then are carriers, or paratenic hosts, of the parasite and able to transmit the infectious larvae to other hosts [59]. Accidental ingestion of this flatworm, recently identified in Florida, USA [60] in considered an important means of transmission for *A*. *cantonensis* in humans, especially in Okinawa, Japan [59, 45]. Other known paratenic hosts include freshwater prawns [61] and frogs [10, 62].

Many physicians and veterinarians are not aware of this parasite or its presence in the United States, and many infections may be misdiagnosed. Human and veterinary medicine would benefit from better, more reliable diagnostics tests, especially in areas with a high prevalence of *A*. *cantonensis*. Since accidental ingestion of the gastropod is a common means of infection, additional research regarding the ability of infectious larvae to survive outside the intermediate gastropod host, either in the mucus, in water or on vegetation, to prevent new cases of angiostrongyliasis is essential. Knowing that *A*. *cantonensis* is present in much of the state of Florida, we can begin to investigate the relationship this parasite has with its gastropod host as well as any potential new rodent and paratenic hosts. The ability for this historically subtropical nematode to maintain itself in hosts in a more temperate climate is alarming and veterinarians and physicians should consider angiostrongyliasis when patients present with unspecified neurological signs. A greater awareness of the prevalence and modes of transmission of *A*. *cantonensis* in the United States may aid in timely diagnosis and ultimately in the prevention of accidental infections.

## Supporting information

S1 File Sequences generated from AcanITS1F1 and AcanITS1R1 standard PCR. A file of generated sequences (*Angiostrongylus cantonensis*) from all real time and standard PCR positive rat, environmental feces and gastropod specimens collected in Florida.
(FASTA)
